# Exosomal HMGB1 Orchestrates NSCLC Progression and Immunosuppressive Macrophage Polarisation Through the TLR4/NF‐κB/IL‐6/STAT3 Signalling Cascade

**DOI:** 10.1111/jcmm.71050

**Published:** 2026-02-06

**Authors:** Jia‐Ru Huang, Wen‐Chao Gu, Ya‐Ping Yuan, Jun‐Xia Yang, Yan Chen, Xiao‐Xia Guo, Wei Ding

**Affiliations:** ^1^ Department of Respiratory and Critical Care Medicine, Shanghai Pudong New Area People's Hospital Shanghai City China; ^2^ Department of Respiratory and Critical Care Medicine, Gongli Hospital of Shanghai Pudong New Area Shanghai City China

**Keywords:** HMGB1, IL‐6, macrophage, NSCLC, STAT3, TLR4/NF‐κB

## Abstract

High mobility group box 1 (HMGB1), a prototypical alarmin and chromatin‐binding protein, has emerged as a critical mediator of tumour‐associated inflammation and immune regulation. Although its soluble form has been implicated in various malignancies, the functional contribution of HMGB1 encapsulated within exosomes remains incompletely understood, particularly in the context of non‐small–cell lung cancer (NSCLC). We profiled exosomal HMGB1 levels in the peripheral blood of 80 clinically annotated NSCLC patients and correlated its abundance with metastatic burden and survival outcomes. Functional experiments using HMGB1‐overexpressing NSCLC cell lines were conducted to assess proliferative, migratory and stemness‐associated phenotypes in vitro, alongside tumorigenicity and drug responsiveness in vivo. Mechanistic interrogation of the TLR4/NF‐κB/IL‐6/STAT3 signalling axis was performed via western blotting, ELISA, immunofluorescence and targeted pharmacologic inhibition. The impact of exosomal HMGB1 on macrophage plasticity was evaluated using THP‐1‐derived macrophage models, and therapeutic relevance was validated in murine tumour models under immunotherapy and chemotherapy regimens. Circulating exosomal HMGB1 levels were significantly elevated in patients with metastatic NSCLC and strongly correlated with poor prognosis. Exosomal HMGB1 markedly enhanced tumour cell proliferation, motility and self‐renewal capacity, while promoting chemoresistance and immune evasion. Mechanistically, HMGB1‐enriched exosomes activated the TLR4/NF‐κB axis, elevating IL‐6 secretion and subsequent STAT3 phosphorylation. These effects were further linked to the polarisation of macrophages towards an immunosuppressive M2 phenotype. Therapeutically, cotargeting STAT3 signalling overcame exosomal HMGB1–mediated resistance to paclitaxel in vivo. Our findings delineate a previously unrecognised exosome‐mediated mechanism by which HMGB1 drives NSCLC progression and modulates the tumour immune microenvironment. Exosomal HMGB1 not only serves as a potential prognostic biomarker but also represents a tractable target for enhancing the efficacy of immuno‐ and chemotherapeutic strategies in NSCLC.

## 1 Introduction

Lung cancer remains the most lethal malignancy globally, with non‐small–cell lung cancer (NSCLC) accounting for approximately 85% of all cases [1, 2]. Despite recent breakthroughs in molecularly targeted therapies and immune checkpoint inhibitors, the clinical outcome of advanced or metastatic NSCLC remains unsatisfactory, primarily due to intrinsic and acquired resistance mechanisms [3, 4, 5]. The complex interplay between tumour cells and the tumour microenvironment (TME) is increasingly recognised as a critical driver of disease progression and therapeutic failure [6, 7].

Exosomes, nanoscale extracellular vesicles (30–150 nm in diameter), have emerged as pivotal mediators of intercellular communication, orchestrating multiple oncogenic processes through the horizontal transfer of bioactive molecules, including nucleic acids, proteins and lipids [8, 9]. Tumour‐derived exosomes (TDEs), in particular, are enriched with tumour‐promoting cargos that can reprogram surrounding stromal and immune cells, enhance metastatic capacity and modulate antitumor immunity [10, 11]. However, the specific molecular composition of exosomes in NSCLC and their impact on metastatic dissemination and immune evasion remain incompletely characterised.

High mobility group box 1 (HMGB1) is a multifunctional DNA‐binding protein that, upon release into the extracellular milieu, functions as a prototypical damage‐associated molecular pattern (DAMP) [12]. HMGB1 can engage pattern recognition receptors such as TLR4 and RAGE, triggering a cascade of pro‐inflammatory and pro‐tumorigenic signalling events [13, 14]. Elevated expression of HMGB1 has been implicated in multiple malignancies and is associated with poor clinical prognosis, increased stemness and resistance to cytotoxic agents [15]. Notably, while extracellular HMGB1 has been extensively studied in the context of sterile inflammation and immunity [16, 17, 18], its role as an exosomal cargo molecule in NSCLC progression has not been systematically interrogated.

Macrophages represent a highly plastic immune subset within the TME and can be polarised towards either a pro‐inflammatory M1 phenotype or an immunosuppressive M2 phenotype [19]. M2‐like tumour‐associated macrophages (TAMs) facilitate tumour growth, angiogenesis and immune suppression [20]. Emerging evidence suggests that tumour‐derived exosomes can educate monocytes and macrophages to acquire a pro‐tumorigenic M2 phenotype [21], yet the molecular determinants of this crosstalk, particularly in the context of NSCLC, remain undefined.

In this study, we identify exosomal HMGB1 as a key mediator linking NSCLC progression with immune modulation. We comprehensively analysed the levels of exosomal HMGB1 in clinical samples and examined its functional relevance using both in vitro and in vivo models. In parallel, we investigated the downstream signalling pathways involved in exosomal HMGB1–mediated tumour–immune crosstalk, with a particular focus on macrophage polarisation and resistance to therapy. Our findings reveal a novel mechanism by which tumour‐derived exosomes orchestrate NSCLC aggressiveness through HMGB1‐dependent immunomodulation and signal activation. These insights not only enhance our understanding of the molecular underpinnings of lung cancer progression but also propose exosomal HMGB1 as a promising diagnostic biomarker and therapeutic target for improving treatment outcomes in NSCLC.

## 2 Materials and Methods

### 2.1 Patient Samples and Exosome Isolation

Peripheral blood samples were obtained from 80 patients with pathologically confirmed NSCLC at Shanghai Pudong New Area People's Hospital prior to treatment. Patients were divided into metastatic (*n* = 32) and nonmetastatic (*n* = 48) groups. Patients were stratified into HMGB1‐low and HMGB1‐high groups based on the median expression level of HMGB1 in the cohort. Blood was processed within 2 h of collection. Plasma was separated by centrifugation at 3000 × g for 15 min at 4°C and stored at −80°C. Exosomes were isolated using a standard ultracentrifugation protocol. Briefly, plasma was subjected to differential centrifugation (2000 × g for 20 min, followed by 10,000 × g for 30 min) to remove cell debris, then ultracentrifuged at 100,000 × g for 90 min. The exosome pellet was washed with PBS and recentrifuged at 100,000 × g. The final pellet was resuspended in PBS for downstream experiments. Protein concentration was determined using the BCA assay (Thermo Fisher Scientific, USA). Exosome size distribution and concentration were analysed using Nanoparticle Tracking Analysis on a NanoSight NS300 system (Malvern Instruments, USA). Particle size (mode and mean diameter) and particle number (particles/ml) were used to standardise treatment dosages. Flow cytometry–based exosome quantification was also performed using the Exo‐Flow Exosome Capture kit (System Biosciences, USA) with CD63‐coated magnetic beads and PE‐labelled antibodies, according to manufacturer instructions. Western blotting confirmed the presence of exosomal markers CD63, CD81 and TSG101.

### 2.2 Cell Culture and Reagents

Human NSCLC cell lines (A549, PC9), human embryonic kidney cells (HEK293T) and the human monocytic leukaemia cell line THP‐1 were obtained from the American Type Culture Collection (ATCC, USA). A549 and PC9 were cultured in RPMI‐1640 (Gibco, USA) with 10% foetal bovine serum (FBS, Gibco, USA). HEK293T cells were cultured in DMEM (Gibco, USA) with 10% FBS. THP‐1 cells were cultured in RPMI‐1640 supplemented with 10% FBS, 1% penicillin–streptomycin and maintained in suspension at 37°C in 5% CO_2_. All cell lines were regularly tested for mycoplasma contamination. IL‐6 neutralising antibodies were obtained from Sino Biological (China). NF‐κB‐IN‐3 (NF‐κB inhibitor, HY‐144744, MedChemExpress, USA) and STAT3‐IN‐3 (STAT3 inhibitor, HY‐128588, MedChemExpress, USA) were obtained from MedChemExpress.

### 2.3 Stable Cell Line Construction

Lentiviral vectors encoding full‐length HMGB1 or empty control were cotransfected with packaging plasmids into HEK293T cells using Lipofectamine 3000 (Thermo Fisher Scientific, USA). Viral supernatants were collected, filtered (0.45 μm, Corning, USA) and used to infect A549 and PC9 cells with 8 μg/mL polybrene. Stable cell lines were selected using puromycin (1 μg/mL) and validated by western blotting for HMGB1 expression.

### 2.4 Exosome Isolation From Cultured Cells

Conditioned medium was collected from cells cultured in exosome‐depleted FBS medium (prepared by ultracentrifugation at 120,000 × g for 16 h). After 48 h, supernatants were sequentially centrifuged (2000 × g, 20 min; 10,000 × g, 30 min), filtered through 0.22 μm filters and ultracentrifuged at 100,000 × g for 90 min. Pellets were washed with PBS, recentrifuged and resuspended in PBS.

### 2.5 Exosome Lysis and Enzyme‐Linked Immunosorbent Assays (ELISA)

To quantify exosome‐contained cytokines, purified exosomes were lysed using RIPA buffer (Beyotime, China) supplemented with protease inhibitors. Lysates were incubated on ice for 30 min, vortexed every 10 min and centrifuged at 12,000 × g for 15 min to remove debris. Supernatants were collected for cytokine quantification. ELISAs were performed using commercial kits for HMGB1 (Thermo Fisher Scientific, USA), Galectin‐9 (R&D Systems, USA), MMP9 (Abcam, UK) and TGF‐β1 (R&D Systems, USA), following manufacturers' protocols. Additionally, cytokine levels in the supernatants of treated THP‐1‐derived macrophages were assessed using the human IL‐6 ELISA kit (R&D Systems, USA), human TNF‐α (R&D Systems, USA) and human IL‐10 (R&D Systems, USA).

### 2.6 Macrophage Polarisation Assay

THP‐1 cells were differentiated into M0 macrophages using 100 ng/mL PMA (Sigma, USA) for 48 h, followed by recovery in PMA‐free medium for 24 h. Peripheral blood mononuclear cells (PBMCs) were isolated from healthy donors using Ficoll‐Paque PLUS (GE Healthcare) after obtaining informed consent and institutional ethical approval. CD14^+^ monocytes were purified by magnetic sorting (Miltenyi Biotec) and differentiated into M0 macrophages by culturing in RPMI‐1640 medium supplemented with 10% FBS, 1% penicillin/streptomycin and recombinant human M‐CSF (50 ng/mL, PeproTech) for 7 days. Macrophages were then treated with exosomes (macrophages: exosomes, 1:10) or recombinant HMGB1 (PeproTech, USA, 100 ng/mL) for 48 h. Total RNA was extracted, and qRT‐PCR or western blotting was performed to evaluate M1 markers (CD80, CD86, iNOS) and M2 markers (CD206, IL‐10, Arg1).

### 2.7 Western Blotting

Cells or exosomes were lysed using RIPA buffer supplemented with protease and phosphatase inhibitors (Beyotime, China). Protein concentrations were quantified via BCA assay (Thermo Fisher Scientific, USA). Equal amounts of protein were resolved by SDS‐PAGE and transferred onto PVDF membranes (Millipore, USA). Membranes were blocked in 5% nonfat milk for 1 h and incubated overnight at 4°C with primary antibodies against HMGB1 (ab18256, Abcam, UK), p‐NF‐κB p65 (phosphor S536, ab76302, Abcam, UK), p‐STAT3 (phosphor Y705, ab267373, Abcam, UK), t‐STAT3 (ab68153, Abcam, UK), p‐JAK2 (phosphor Y1007, ab195055, Abcam, UK), t‐JAK2 (ab108596, Abcam, UK), anti‐CD206 (ab64693, Abcam, UK), anti‐CD86 (ab239075, Abcam, UK). After washing, membranes were incubated with HRP‐conjugated secondary antibodies (1:5000) for 1 h at room temperature and visualised using ECL reagents (Bio‐Rad).

### 2.8 Immunohistochemistry (IHC)

Paraffin‐embedded NSCLC tumour tissues were sectioned (4 μm), deparaffinised and rehydrated. Antigen retrieval was performed using sodium citrate buffer (pH 6.0) in a microwave. Endogenous peroxidase activity was blocked with 3% H₂O₂. Slides were incubated with anti‐HMGB1 antibody (1:200, ab18256, Abcam, UK) overnight at 4°C, followed by HRP‐conjugated secondary antibodies and DAB substrate (Invitrogen, USA). Counterstaining was performed with haematoxylin. Staining intensity was scored independently by two pathologists.

### 2.9 Immunofluorescence Staining

Cells were fixed in 4% paraformaldehyde, permeabilised with 0.2% Triton X‐100, blocked with 5% BSA (Invitrogen, USA) and incubated with primary antibodies against p‐STAT3 (ab267373, Abcam, UK) overnight at 4°C. After washing, cells were incubated with Alexa Fluor‐conjugated secondary antibodies (Invitrogen, USA) for 1 h and counterstained with DAPI (Invitrogen, USA). Images were captured using a confocal microscope (Zeiss LSM 880, Germany).

### 2.10 qRT‐PCR

Total RNA was extracted using TRIzol reagent (Invitrogen, USA), and cDNA was synthesised using a reverse transcription kit (Takara, Japan). Quantitative PCR was performed using SYBR Green Master Mix (Vazyme, USA) on a CFX96 real‐time PCR system (Bio‐Rad, USA). Relative gene expression was calculated using the 2^−^ΔΔCt method, normalised to GAPDH. Primer sequences are downloaded from Primerbank.

### 2.11 Cell Viability and Functional Assays

Cell proliferation was measured using the CCK‐8 assay and complemented by flow cytometric cell counting, thereby reducing the potential confounding effects of altered cellular metabolism. Transwell migration assays were performed in serum‐free medium using 8 μm pore‐size inserts. Colony formation was assessed by culturing 500 cells per well for 5 days in 3D Matrigel gels (Coring, USA, 5 mg/mL). Apoptosis was evaluated by Annexin V‐FITC/PI staining and flow cytometry (Becton & Dickinson, USA) after 48 h of treatment with paclitaxel (20 nM, Sangon, China), cisplatin (5 μg/mL, Sigma, USA) or osimertinib (100 nM, Sigma, USA).

### 2.12 Animal Experiments

All animal procedures were approved by the Institutional Animal Care and Use Committee of Shanghai Pudong New Area People's Hospital & Shanghai Pudong New Area Gongli Hospital (2025‐D‐96). Female NOD‐SCID mice (4–6 weeks old) were subcutaneously injected with 5 × 10^6^ A549 cells. After tumour volume reached ~100 mm^3^, mice were randomly assigned to receive intratumour injections of PBS, recombinant HMGB1 (10 or 100 ng/mouse) or exosomes (1 × 10^10^ particles/mouse) twice weekly. For drug resistance assays, mice were treated with paclitaxel (10 mg/kg, i.p., twice a week), with or without STAT3 inhibitor (STAT3‐IN‐3, 5 mg/kg, i.p., MedChemExpress, USA). Tumour volumes were measured every 3 days using callipers, and volume was calculated as (length × width^2^)/2. For immune therapy studies, C57BL/6 mice were implanted with Lewis lung carcinoma cells (1 × 10^6^ cells/mouse). When tumours reached ~80 mm^3^, mice were treated with exosomes and/or anti‐PD‐1 antibody (RMP1‐14, 200 μg/mouse, MedChemExpress, USA) intraperitoneally, twice weekly. Tumour growth and response were monitored, and mice were euthanised when the tumour diameter exceeded 15 mm.

### 2.13 Bioinformatic Analyses

Gene expression, survival and immune infiltration analyses were performed using data from TCGA, GEO (GSE72094, GSE30219), Sangerbox (http://sangerbox.com) and GEPIA2 platforms. Stemness correlation analysis was conducted using the stemness score derived from Sangerbox (http://sangerbox.com). Protein–protein interaction (PPI) networks were constructed using the STRING database (https://string‐db.org/). The minimum required interaction score was set to 0.4 (medium confidence), and both direct (physical) and indirect (functional) associations were included. Only interactions supported by experimental evidence, curated databases or coexpression data were considered. Network visualisation and further analysis were performed using the default settings in STRING.

### 2.14 Statistical Analysis

Data are presented as mean ± SD from at least three independent experiments. Statistical significance was assessed using Student's *t*‐test or one‐way ANOVA followed by Tukey's post hoc test. Kaplan–Meier survival curves were analysed by the log‐rank test. Correlations were assessed using Pearson's coefficient. *p* < 0.05 was considered statistically significant. Analyses were performed using GraphPad Prism 9.0.

## 3 Results

### 3.1 High Expression of Exosomal HMGB1 Predicts Poor Prognosis in NSCLC Patients

**FIGURE 1:**
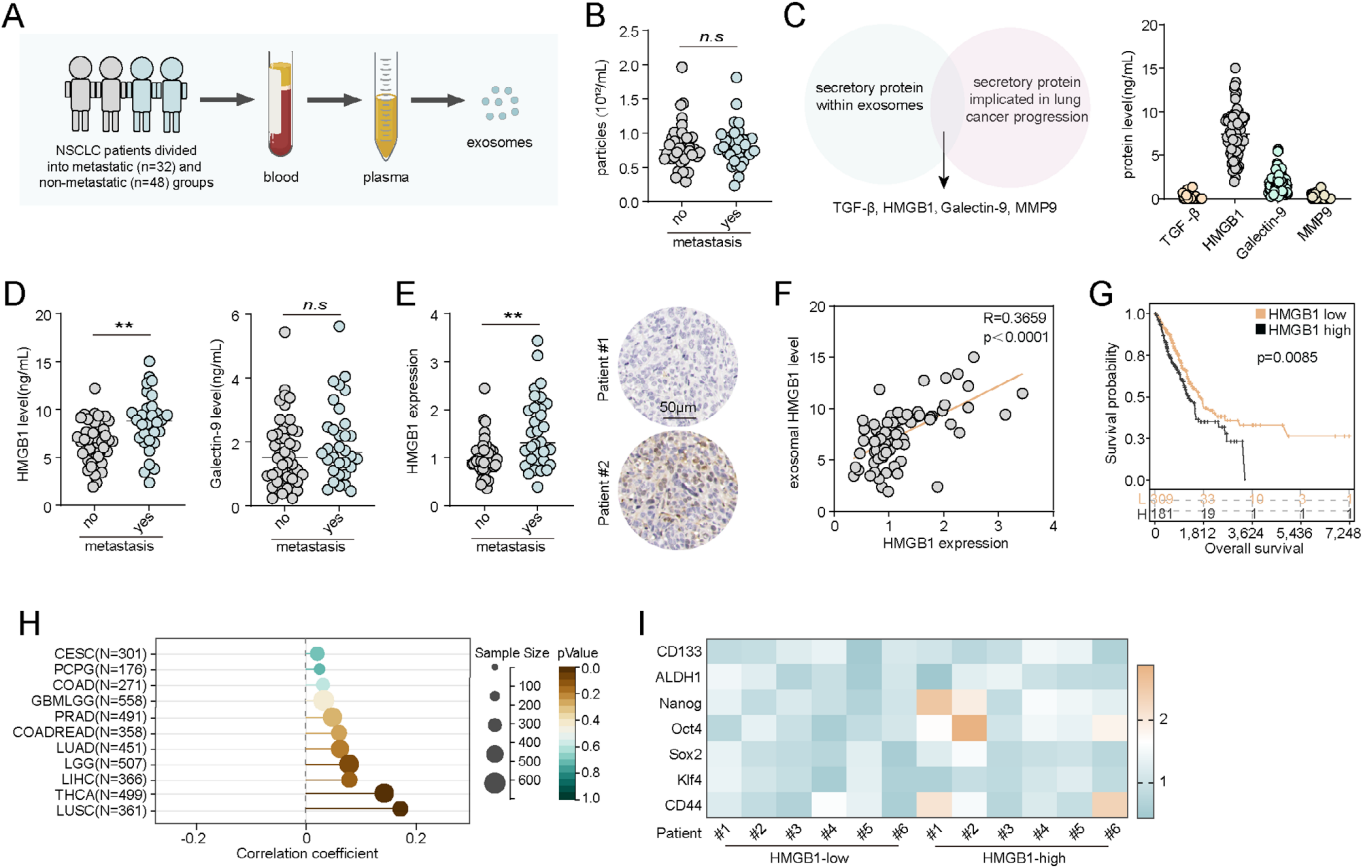
Exosomal HMGB1 predicts poor prognosis in NSCLC. (A) A total of 80 NSCLC patients were categorised into metastasis (*n* = 32) and nonmetastasis (*n* = 48) groups. Blood‐derived exosomes were isolated, quantified and subjected to protein extraction for further analysis. A schematic workflow is presented. (B) Quantification of circulating exosome numbers in blood samples from the metastasis and nonmetastasis NSCLC patient groups. (C) Potential secretory protein factors within exosomes, particularly those implicated in lung cancer progression, were analysed. HMGB1 and Galectin‐9 was identified as a key candidate. A schematic diagram and quantification of exosomal TGF‐β, HMGB1, Galectin‐9 and MMP9 are shown. (D) Quantification of exosomal HMGB1 and galectin‐9 levels in the metastasis and nonmetastasis NSCLC groups. (E) Immunohistochemical (IHC) staining of HMGB1 in tumour tissues from patients with and without metastasis, followed by statistical analysis. (F) Correlation analysis between circulating exosomal HMGB1 concentrations and HMGB1 expression in tumour tissues from all 80 NSCLC patients. (G) Kaplan–Meier survival analysis based on HMGB1 expression in 558 NSCLC patients from the TCGA database. (H) Correlation analysis between HMGB1 expression and stemness scores across different cancer types using the Sanger database. (I) Heatmap showing expression of core stemness‐related genes (CD133, ALDH1, Nanog, Oct4, SOX2, KLF4, CD44) in tumour tissues from 12 NSCLC patients, stratified into HMGB1^low^ and HMGB1^high^ groups.

To evaluate whether the distribution of peripheral blood‐derived exosomes is associated with distant metastasis in NSCLC, we first collected blood samples from 80 NSCLC patients. Based on clinicopathological characteristics, patients were divided into metastatic (*n* = 32) and nonmetastatic (*n* = 48) groups. Exosomes were isolated from plasma via ultracentrifugation and subjected to quantification and protein extraction. A schematic diagram of the experimental workflow was illustrated (Figure 1A). The results showed that the number of circulating exosomes did not significantly differ between metastatic and nonmetastatic groups (Figure 1B), suggesting that the biological content of exosomes, rather than their abundance, might be the key factor influencing tumour behaviour. We subsequently screened and analysed several secretory factors that are known to be involved in lung cancer progression and have been previously reported to be encapsulated in exosomes, including TGF‐β, HMGB1, Galectin‐9 and MMP9. We found that exosomes isolated from NSCLC patient plasma contained detectable levels of HMGB1 and Galectin‐9 (Figure 1C). Quantitative analysis of exosomal Galectin‐9 and HMGB1 in all patient samples revealed that HMGB1 levels were significantly elevated in exosomes from the metastatic group compared to the nonmetastatic group (Figure 1D). To further validate the expression of HMGB1 at the tissue level, we performed immunohistochemical analysis of tumour tissues and found a significant upregulation of HMGB1 expression in the metastatic group (Figure 1E). Moreover, a strong positive correlation was observed between exosomal HMGB1 levels and HMGB1 expression in tumour tissues (Figure 1F), suggesting that exosomal HMGB1 may reflect tumour‐derived HMGB1 and potentially contribute to tumour progression. Survival analysis using the TCGA database of 558 NSCLC patients revealed that high HMGB1 expression was associated with significantly worse overall survival (Figure 1G). Given that HMGB1 may promote metastasis by enhancing tumour stemness, we further analysed the correlation between HMGB1 expression and stemness scores across multiple cancer types using the Sanger database. A positive correlation was observed in various cancers, particularly lung cancer (Figure 1H). Additionally, in 12 NSCLC tumour samples, we profiled the expression of stemness‐associated genes (CD133, ALDH1, Nanog, Oct4, SOX2, KLF4 and CD44) and found that HMGB1‐high tumours exhibited a general upregulation of stemness gene expression, especially Nanog, Oct4 and CD44 (Figure 1I). These findings suggest that high exosomal HMGB1 expression is closely associated with distant metastasis and poor prognosis in NSCLC patients.

### 3.2 Exosomal HMGB1 Promotes Malignant Biological Behaviour of NSCLC Cells

**FIGURE 2:**
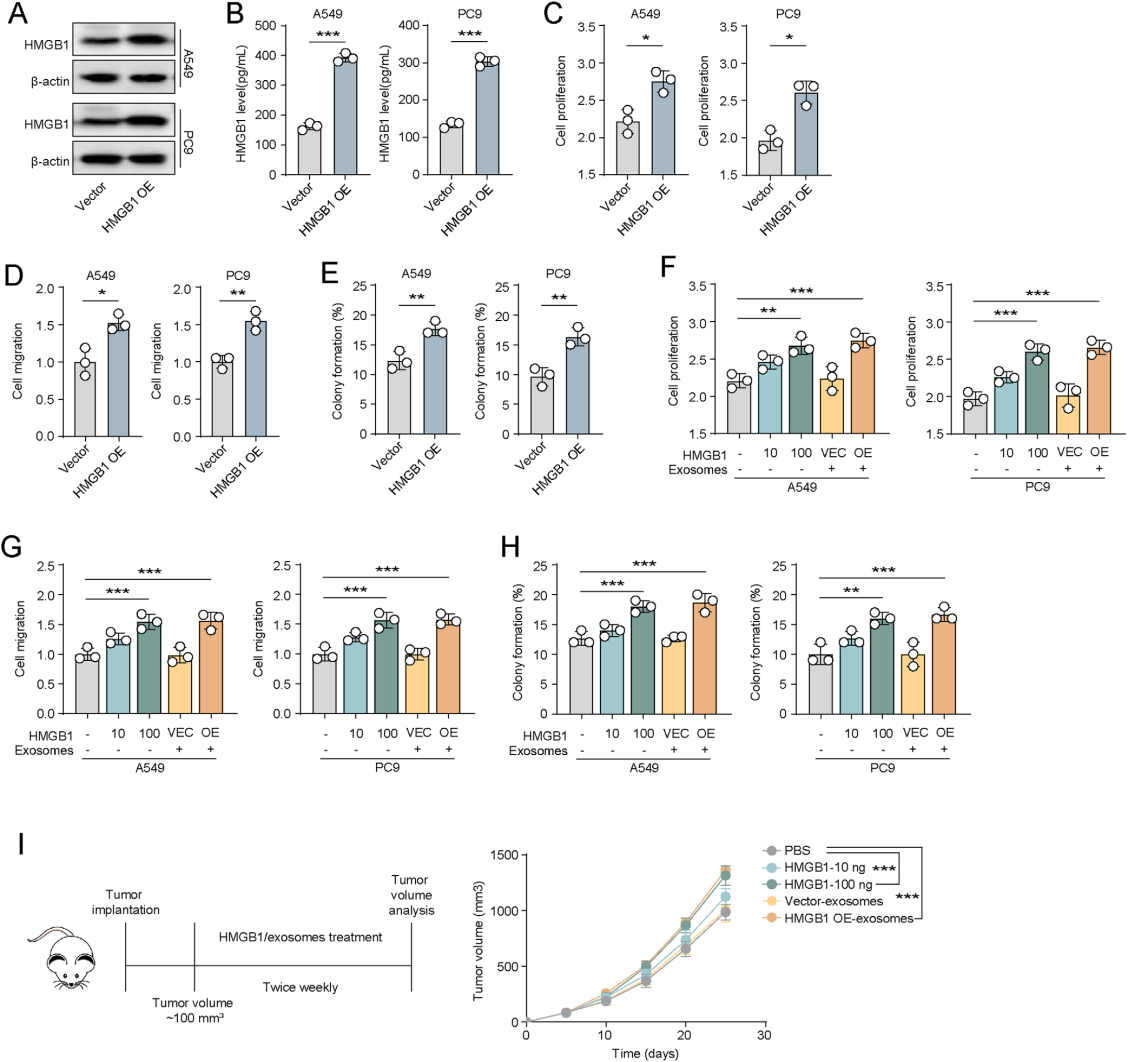
Exosomal HMGB1 promotes NSCLC progression. (A) Western blot analysis of HMGB1 expression in vector control and HMGB1‐overexpressing (OE) A549 and PC9 cells. (B) Measurement of HMGB1 levels in exosomes derived from vector control and HMGB1 OE A549 and PC9 cells. (C) Cell proliferation assays of vector and HMGB1 OE A549 and PC9 cells. (D) Cell migration assays in vector and HMGB1 OE A549 and PC9 cells. (E) Colony formation assays for vector and HMGB1 OE A549 and PC9 cells. (F) Cell proliferation of A549 and PC9 cells treated with PBS, recombinant HMGB1 (10 or 100 ng) or exosomes derived from vector or HMGB1 OE cells (cell‐to‐exosome ratio = 1:10). (G) Cell migration of A549 and PC9 cells under the same treatment conditions as in (F). (H) Colony formation capacity of A549 and PC9 cells under the same treatment conditions as in (F). (I) Tumour volume in A549‐bearing mice treated with PBS, HMGB1 (10 ng or 100 ng per mouse) or exosomes from vector or HMGB1 OE cells (1 × 10^10^ exosomes per mouse). (J) A549 and PC9 cells were seeded at densities of 1 × 10^5^, 1 × 10^6^ and 5 × 10^6^ cells per six‐well plate and cultured for 3 days. Exosome production was then analysed.

To further investigate the impact of exosomal HMGB1 on NSCLC cell behaviour, we established A549 and PC9 cell lines with stable HMGB1 overexpression (OE) and corresponding vector controls. Western blot analysis confirmed significantly increased HMGB1 expression in HMGB1 OE cells (Figure 2A), and a concomitant elevation of HMGB1 levels in exosomes derived from these cells (Figure 2B). Functional assays revealed that HMGB1 OE cells exhibited enhanced proliferation (Figure 2C), migration (Figure 2D) and colony‐forming abilities (Figure 2E) compared to controls. To determine whether HMGB1 exerts its pro‐tumorigenic effects in a secreted or exosomal form, we treated A549 and PC9 cells with recombinant HMGB1 protein (10 ng or 100 ng) or with exosomes derived from vector or HMGB1 OE cells (applied at a 1:10 cell‐to‐exosome ratio). Both recombinant HMGB1 and HMGB1 OE‐derived exosomes significantly enhanced cell proliferation (Figure 2F), migration (Figure 2G) and colony formation (Figure 2H). Notably, HMGB1 OE‐derived exosomes showed stronger tumour‐promoting effects, whereas the low dose of free HMGB1 (10 ng) did not significantly promote malignancy, even though the amount of HMGB1 contained in the exosomes was less than 10 ng. Additionally, treatment of HMGB1‐IN1 (a HMGB1 inhibitor) significantly attenuated the exosomal HMGB1–mediated pro‐proliferative and pro‐migratory effects. Treatment with the HMGB1 inhibitor alone had minimal effects on tumour cell proliferation, migration and colony formation (Figure S1A–C). This suggests that exosomal encapsulation enhances cellular uptake of HMGB1 through endocytosis, resulting in a more pronounced biological effect. In vivo experiments further validated these findings. In A549 xenograft‐bearing mice, administration of HMGB1 OE‐derived exosomes (1 × 10^10^ particles/mouse) or recombinant HMGB1 (100 ng/mouse) significantly promoted tumour growth, while low‐dose HMGB1 (10 ng/mouse) had minimal effects (Figure 2I). To explore why tumours utilise exosomes to modulate tumour progression, we assessed exosome production under different cell densities. A549 and PC9 cells were cultured at varying densities (1 × 10^5^, 1 × 10^6^ and 5 × 10^6^ cells/well), and exosome yield was measured after 3 days. The results showed that higher cell density induced greater exosome secretion (Figure S2A), suggesting that tumour cells may release membrane vesicles, such as exosomes, as a programmed response to overgrowth stress. Exosomal HMGB1, in turn, may accelerate systemic tumour progression by enhancing cell migration and stemness. Collectively, these results demonstrate that exosomal HMGB1 promotes malignant behaviour in NSCLC cells.

### 3.3 Exosomal HMGB1 Promotes NSCLC Progression via TLR4/NF‐κB/IL‐6/STAT3 Signalling Pathway

**FIGURE 3:**
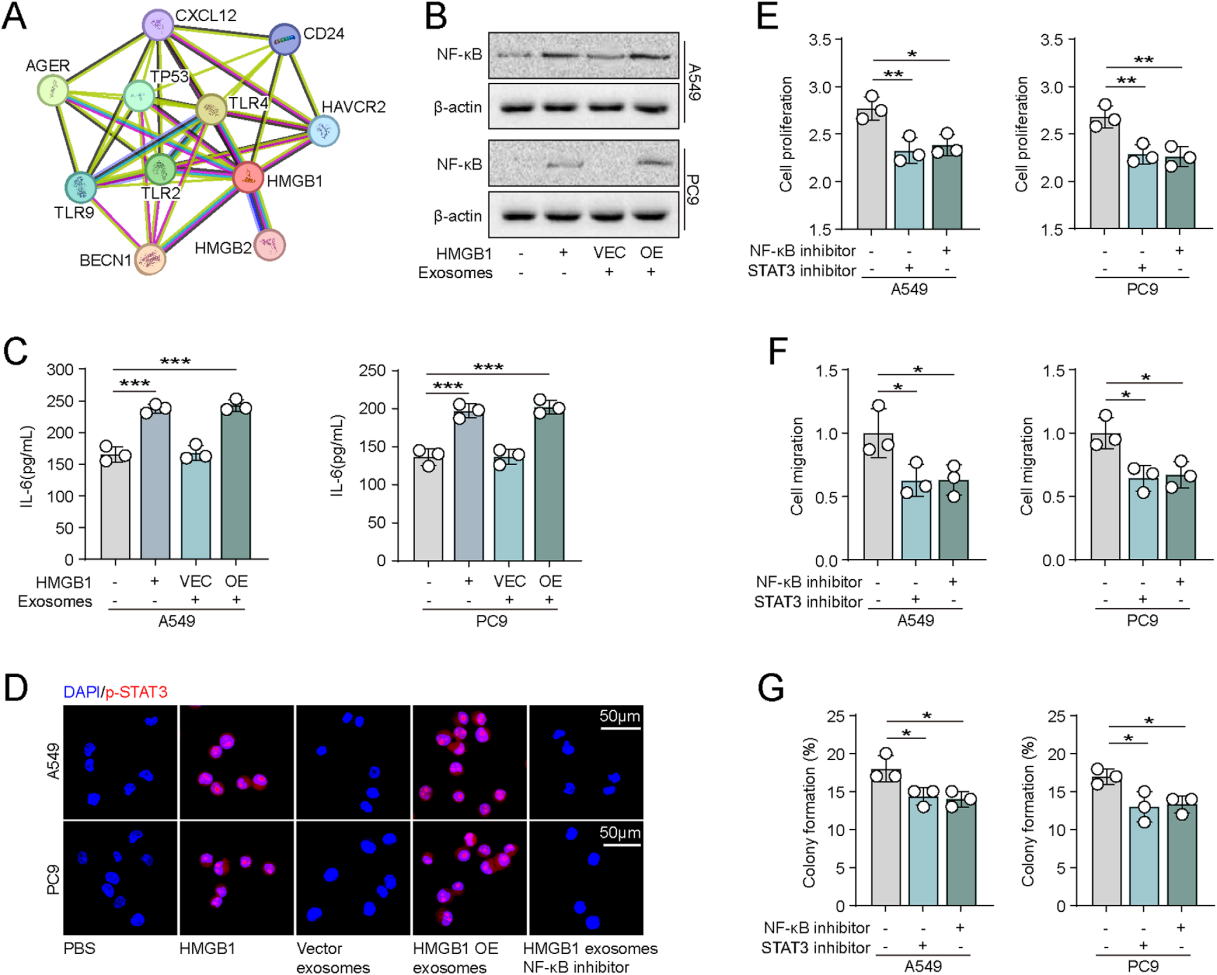
Exosomal HMGB1 activates JAK/STAT3 signalling to promote NSCLC progression. (A) Protein–protein interaction (PPI) network analysis of HMGB1 using the STRING database. (B) Western blot analysis of NF‐κB in A549 and PC9 cells treated with PBS, recombinant HMGB1 (100 ng), exosomes from vector cells or exosomes from HMGB1 OE cells (cell‐to‐exosome ratio = 1:10). (C) ELISA quantification of IL‐6 in the supernatant of A549 and PC9 cells under the same treatment conditions as in (B). (D) Immunofluorescence staining of p‐STAT3 of A549 and PC9 cells under the same treatments, including an additional group co‐treated with exosomes from HMGB1 OE cells and NF‐κB inhibitor (50 μM). (E) Cell proliferation of A549 and PC9 cells treated with HMGB1 OE‐derived exosomes alone or in combination with NF‐κB inhibitor (50 μM) or STAT3 inhibitor (20 μM). (F) Cell migration under the same treatment conditions as in (E). (G) Colony formation assays of A549 and PC9 cells under the same treatment conditions as in (E).

To elucidate the molecular mechanisms underlying HMGB1‐driven tumour progression, we performed a protein–protein interaction (PPI) analysis using the STRING database. The results indicated a potential interaction between HMGB1 and TLR4 (Figure 3A). TLR4 is known to activate the canonical NF‐κB/IL‐6 signalling axis [22], which plays a critical role in tumour growth. Accordingly, we treated A549 and PC9 cells with PBS, recombinant HMGB1 (100 ng), vector‐derived exosomes or HMGB1 OE‐derived exosomes. Western blot analysis revealed that both HMGB1 and HMGB1 OE exosomes markedly increased NF‐κB activation (Figure 3B). Blockade of TLR4 by siRNAs also suppressed the NF‐κB activation in A549/PC9 cells treated with exosomal HMGB1 (Figure S2B,C). Consistently, ELISA showed a significant elevation of IL‐6 in the culture supernatants of cells treated with HMGB1 OE exosomes (Figure 3C). As IL‐6 is a potent activator of the JAK2/STAT3 pathway in tumours [23], immunofluorescence staining further demonstrated that HMGB1 OE exosomes significantly increased the phosphorylation of STAT3 (p‐STAT3), which was attenuated upon treatment with the NF‐κB inhibitor NF‐Κb‐IN‐3 (50 μM) (Figure 3D). Meanwhile, treatment of IL‐6 neutralising antibodies suppressed the activation of STAT3 in HMGB1 OE exosomes cocultured with A549/PC9 cells (Figure S2D). We further investigated the time‐dependent activation of the JAK2/STAT3 signalling pathway in lung cancer cells upon exposure to exosomal HMGB1 (0, 24, 48 and 72 h). The results revealed that exosomal HMGB1 induced a marked activation of JAK2/STAT3 signalling, with peak phosphorylation observed at 48 h (Figure S2E). Functionally, the enhanced cell proliferation (Figure 3E), migration (Figure 3F) and colony formation (Figure 3G) induced by HMGB1 OE exosomes were partially reversed by inhibitors of either NF‐κB or STAT3. These findings suggest that HMGB1 may promote malignant phenotypes by activating the NF‐κB/IL‐6 axis, thereby stimulating the JAK2/STAT3 signalling pathway.

### 3.4 Targeting HMGB1 Signalling Improves Therapeutic Response in NSCLC

**FIGURE 4:**
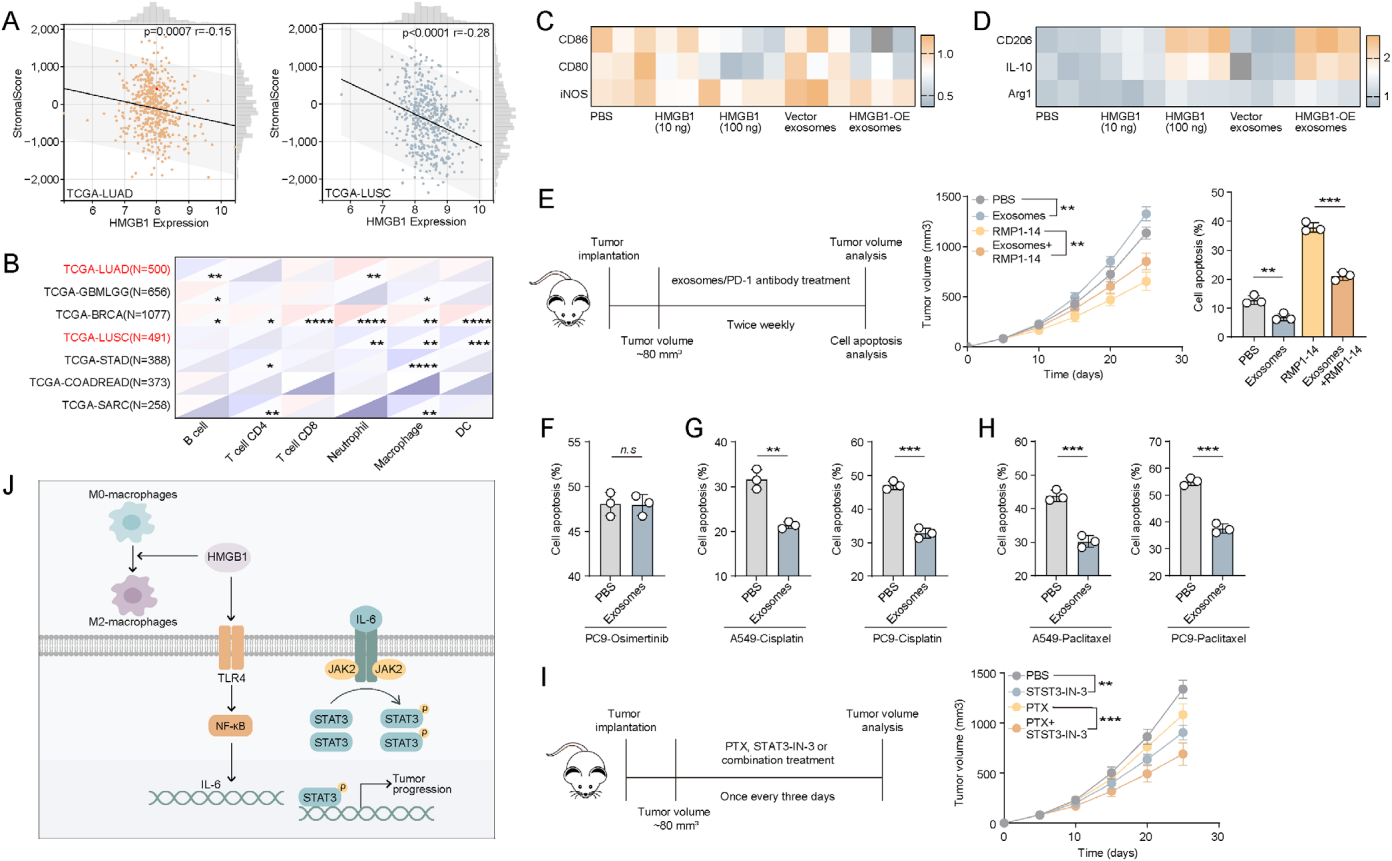
Targeting HMGB1 signalling improves therapeutic outcomes in NSCLC. (A) Correlation analysis between immune infiltration scores and HMGB1 expression in 491 LUAD and 500 LUSC patients from the TCGA database. (B) Correlation between HMGB1 expression and the distribution of various immune cell subsets in LUAD and LUSC patients. (C, D) THP‐1–derived M0 macrophages were treated with PBS, HMGB1 (10 or 100 ng) or exosomes derived from vector or HMGB1 OE cells (cell‐to‐exosome ratio = 1:10). M1 macrophage markers (CD86, CD80, iNOS) and M2 markers (CD206, IL‐10, Arg1) were quantified by PCR. (E) Lewis tumour‐bearing mice were treated with PBS, HMGB1 OE‐derived exosomes (1 × 10^10^ exosomes per mouse, twice per week), anti‐PD‐1 antibody (RMP1‐14, 200 μg per mouse, twice per week) or combination therapy (*n* = 5 per group). Tumour volumes and apoptosis levels in tumour tissues (day 25) were assessed. (F) PC9 cells were treated with PBS or exosomes from HMGB1 OE cells (cell‐to‐exosome ratio = 1:10), followed by Osimertinib (50 nM, 48 h), and apoptosis was measured. (G) A549 and PC9 cells were similarly treated with PBS or HMGB1 OE‐derived exosomes, followed by Cisplatin (5 μM, 48 h), and apoptosis was analysed. (H) A549 and PC9 cells were similarly treated with paclitaxel (10 μM, 48 h) under the same conditions, and cell apoptosis was determined. (I) A549‐bearing mice were treated with HMGB1 OE‐derived exosomes (1 × 10^10^ exosomes per mouse), followed by PBS, paclitaxel (PTX, 10 mg/kg, twice per week), STAT3 inhibitor (5 mg/kg, twice per week) or combination therapy. (J) Schematic diagram illustrating the proposed mechanism: HMGB1 upregulates TLR4, thereby activating the NF‐κB–IL‐6 axis and stimulating JAK2/STAT3 signalling to promote tumour progression. Concurrently, HMGB1 facilitates M2 macrophage polarisation.

To explore the relationship between HMGB1 and the tumour immune microenvironment, we analysed TCGA data from 491 LUAD and 500 LUSC patients. The results showed a potential negative correlation between HMGB1 expression and immune infiltration scores in lung cancer, especially in LUSC (Figure 4A). Further analysis revealed that HMGB1 expression negatively correlated with macrophage infiltration across most tumours, including LUSC (Figure 4B), prompting us to investigate whether HMGB1 affects macrophage polarisation. In vitro, we treated THP‐1‐derived M0 macrophages with PBS, recombinant HMGB1 (10 ng or 100 ng), vector‐derived exosomes or HMGB1 OE‐derived exosomes. qPCR analysis showed that HMGB1 OE exosomes and high‐dose HMGB1 significantly upregulated M2 markers (CD206, IL‐10, Arg1) and downregulated M1 markers (CD86, CD80, iNOS) (Figure 4C,D). Western blotting showed that OE exosomes and high‐dose HMGB1 significantly upregulated CD206 and downregulated CD86 (Figure S2F), indicating a shift towards M2 polarisation. The ELISA assay showed that OE exosomes and high‐dose HMGB1 increased the IL‐10 secretion of IL‐10 of macrophages; however, no difference was found in TNF‐α secretion (Figure S2G), which might be due to the low endogenous expression of TNF‐α in M0 macrophages. Furthermore, M0 macrophages derived from human peripheral blood monocytes were treated with recombinant HMGB1 or exosomes. Consistently, high concentrations of HMGB1 or exosomes from HMGB1‐overexpressing cells markedly promoted M2 polarisation of macrophages (Figure S1H). Notably, exosomes alone had minimal effects on macrophage polarisation. Given the immunomodulatory role of macrophages, we next examined whether exosomal HMGB1 influences immunotherapy efficacy. In a Lewis lung carcinoma mouse model, we administered HMGB1 OE‐derived exosomes and/or anti‐PD‐1 antibody (RMP1‐14). Mice treated with exosomal HMGB1 exhibited reduced sensitivity to PD‐1 blockade, and Annexin V/PI staining showed decreased tumour cell apoptosis in the exosome group (Figure 4E), suggesting that exosomal HMGB1 may contribute to immune evasion and resistance to immunotherapy. We further assessed the impact of exosomal HMGB1 on targeted and chemotherapeutic agents. PC9 cells pretreated with PBS or HMGB1 OE exosomes were subsequently treated with osimertinib, with no observed difference in drug sensitivity (Figure 4F). However, in both PC9 and A549 cells, HMGB1 OE exosomes significantly reduced apoptosis induced by cisplatin or paclitaxel (Figure 4G,H). These results indicate that while EGFR‐targeted therapy remains effective in EGFR‐mutant NSCLC, chemoresistance induced by exosomal HMGB1 should be addressed in EGFR wild‐type patients. Finally, in A549 xenograft‐bearing mice, we treated animals with HMGB1 OE‐derived exosomes followed by paclitaxel with or without a STAT3 inhibitor. Combined therapy significantly suppressed tumour growth, suggesting that HMGB1‐induced drug resistance can be overcome by targeting STAT3 signalling (Figure 4I). These findings underscore the critical role of exosomal HMGB1 in modulating NSCLC cell behaviour and the immune microenvironment and highlight its potential as a diagnostic marker and therapeutic target across distinct patient subgroups.

## 4 Discussion

Exosomes have emerged as critical mediators of intercellular communication in cancer biology, acting as carriers of oncogenic proteins, RNAs and lipids that modulate recipient cell behaviour and remodel TME [24, 25, 26]. Numerous studies have demonstrated that tumour‐derived exosomes can enhance angiogenesis, mediate immune escape and foster premetastatic niche formation. For example, melanoma‐ and breast cancer–derived exosomes have been shown to transfer pro‐metastatic signals to distant organs, priming them for subsequent tumour colonisation [27, 28]. However, while the pro‐tumorigenic functions of exosomes are increasingly appreciated, the identification of specific cargoes responsible for immune reprogramming and metastasis in NSCLC remains incomplete. Our study fills this gap by demonstrating that NSCLC‐derived exosomal HMGB1 is a pivotal molecule that drives macrophage M2 polarisation, activates pro‐oncogenic signalling pathways and promotes tumour growth and chemoresistance.

As previously described, HMGB1, a nuclear protein that can function extracellularly as a damage‐associated molecular pattern, has long been associated with tumour progression, inflammation and immune modulation [12, 15]. Previous studies have shown that secreted HMGB1 contributes to tumour invasion and metastasis by engaging pattern recognition receptors such as TLR4 and RAGE [29, 30, 31]. Nonetheless, most of these investigations rely on supraphysiological concentrations of recombinant HMGB1, which are rarely observed in vivo. Our findings provide a paradigm shift by showing that exosome‐encapsulated HMGB1 exerts significant biological effects at low concentrations, plausibly due to its efficient endocytic uptake by recipient cells. This encapsulation‐based delivery system not only enhances cellular uptake but may also shield HMGB1 from extracellular degradation and receptor antagonism, ensuring its functional preservation. Thus, we propose that exosomal HMGB1 represents the physiologically relevant form through which HMGB1 exerts its tumour‐promoting functions in vivo.

The TLR4/NF‐κB/IL‐6/STAT3 signalling axis, activated downstream of HMGB1 in our study, is a well‐established oncogenic pathway involved in both intrinsic tumour progression and extrinsic immunosuppression [32, 33]. Persistent activation of STAT3 has been linked to tumour stemness, drug resistance and immune evasion in various cancers, including NSCLC [34, 35, 36]. While prior studies have described TLR4/NF‐κB activation by HMGB1 in immune cells or tumour cells separately, our work is among the first to connect exosomal HMGB1 to coordinated activation of this pathway in both compartments. Specifically, we show that exosomal HMGB1 reprograms macrophages towards an immunosuppressive M2 phenotype via this cascade, while simultaneously enhancing NSCLC cell malignancy and resistance to paclitaxel. The fact that STAT3 inhibition reverses exosome‐mediated resistance underscores its central role and offers a translationally actionable target.

Macrophages represent a dominant immune cell population in the TME and are known to adopt pro‐ or antitumoral phenotypes depending on contextual cues [37]. While multiple studies have shown that tumour‐secreted factors can polarise macrophages into an M2‐like state, the specific role of tumour‐derived exosomes in this process is still being elucidated [38]. Recent research has suggested that tumour exosomes may carry miRNAs (e.g., miR‐21, miR‐1246) or proteins that induce M2 polarisation, yet few studies have pinpointed individual protein cargoes with such activity [10, 39]. Here, we identify HMGB1 as a key exosomal driver of macrophage M2 polarisation, directly linking tumour‐derived exosomes to immune suppression via a defined molecular mechanism. This finding not only identified a functional content of lung tumour exosomes but also provides a specific target for therapeutic intervention.

Another important finding of our study is the density‐dependent release of exosomes by tumour cells, which may reflect an adaptive response to nutrient depletion and spatial constraints within rapidly growing tumours. This phenomenon provides a plausible explanation for how advanced tumours promote their own dissemination: as tumour burden increases, exosome secretion is upregulated, thereby enhancing both local invasiveness and systemic metastasis through immune modulation and niche conditioning. The ability of exosomes to travel through the bloodstream and prime distant sites for metastasis by altering immune composition, particularly macrophage polarisation, underscores their central role in tumour ecology. In contrast to previous studies that often focused on either exosome biology or HMGB1 function in isolation, our research integrates both fields and identifies a specific molecular bridge—exosomal HMGB1—that links tumour cell‐intrinsic signalling, immune remodelling and therapy resistance. The identification of exosomal HMGB1 as a predictive biomarker of metastasis and poor prognosis further strengthens its clinical relevance. Importantly, our findings suggest that therapeutic strategies targeting exosomal packaging or uptake of HMGB1—such as inhibitors of exosome biogenesis or endocytosis—may offer synergistic benefits when combined with STAT3 inhibition or chemotherapy. While our study provides insights into exosomal HMGB1‐mediated NSCLC progression, it has limitations. Subcutaneous xenograft models, though convenient, do not fully reflect the lung tumour microenvironment, and orthotopic models may offer higher clinical relevance. Additionally, all in vivo experiments in this study were performed using female mice, which may limit the generalisability of the findings across sexes. Sex‐specific differences might influence tumour biology, immune cell composition and responses to chemotherapy and immunotherapy, potentially through hormonal regulation and differential activation of inflammatory signalling pathways. Therefore, it remains possible that exosomal HMGB1–mediated tumour–immune interactions may exhibit quantitative or contextual differences in male hosts. Importantly, several aspects of our conclusions remain well supported despite this limitation. The clinical associations between exosomal HMGB1 levels, metastatic burden and patient prognosis were derived from human cohorts without sex restriction. Moreover, the mechanistic framework linking exosomal HMGB1 to TLR4/NF‐κB/IL‐6/STAT3 activation and macrophage M2 polarisation was consistently validated using human NSCLC cell lines and macrophage models in vitro, supporting the biological relevance of this pathway independent of mouse sex. Future studies incorporating both male and female animals, as well as orthotopic lung cancer models, will be required to fully elucidate potential sex‐dependent effects of exosomal HMGB1 on tumour progression, immune regulation and therapeutic response. Next, while our study demonstrates the pro‐tumorigenic role of exosomal HMGB1, we did not perform experiments using HMGB1‐neutralising antibodies to directly block its effects. Such experiments would provide stronger causal evidence, and further studies should be included when suitable reagents become available. Thirdly, although our findings highlight exosomal HMGB1 as a potential prognostic biomarker in NSCLC, its superiority over soluble or tissue‐localised HMGB1 has not been directly established. Public datasets do not yet provide survival outcomes specific to exosomal HMGB1, and prospective validation is ongoing. Nevertheless, we observed a strong correlation between exosomal and total HMGB1 levels, and exosomal HMGB1 was associated with metastatic burden, suggesting its potential utility. Importantly, blood‐based detection of exosomal HMGB1 offers a minimally invasive approach compared with tissue biopsy, which may facilitate clinical translation. Additionally, although we used anti‐PD‐1 antibodies in our immunotherapy experiments to align with standard preclinical models, we recognise that PD‐L1 is expressed on macrophages and may be more directly relevant to the effects of exosomal HMGB1 on macrophage polarisation. Future studies employing anti‐PD‐L1 inhibitors could provide more mechanistic insight. Finally, we did not investigate whether HMGB1 overexpression alters the abundance of other functional proteins in exosomes, nor did we examine posttranslational modifications of HMGB1, which can influence its activity. Comprehensive proteomic analyses will be required in future studies to address these points and clarify the specific contributions of HMGB1 to exosome‐mediated effects.

In summary, this study reveals a novel mechanism by which NSCLC cells exploit exosomal HMGB1 to modulate the immune microenvironment and enhance malignant progression. By elucidating the critical role of exosomal HMGB1 in macrophage polarisation, pro‐tumoral signalling and chemoresistance, our findings not only deepen mechanistic understanding of tumour–immune interactions but also pave the way for the development of novel therapeutic strategies that disrupt this exosome‐mediated communication axis.

## Author Contributions

Jia‐Ru Huang and Wen‐Chao Gu contributed equally to this article. This study was proposed and designed by Jia‐Ru Huang and Wei Ding; Jia‐Ru Huang, Wen‐Chao Gu, and Ya‐Ping Yuan collected patient samples; Jia‐Ru Huang, Wen‐Chao Gu, Ya‐Ping Yuan, and Jun‐Xia Yang conducted the experiments; Jia‐Ru Huang and Wen‐Chao Gu collected and processed raw data; Jia‐Ru Huang, Wen‐Chao Gu, and Yan Chen conducted the bioinformatic and statistical analyses; Jia‐Ru Huang, Wen‐Chao Gu, Ya‐Ping Yuan, Jun‐Xia Yang, Yan Chen, and Xiao‐Xia Guo drew pictures; Jia‐Ru Huang and Wen‐Chao Gu wrote manuscript draft; Jia‐Ru Huang and Wei Ding made revisions. All authors have read and approved this research to be published.

## Funding

This research was funded by the 2024 Pudong New Area Clinical Medicine Specialty Development Program for Peak and Plateau Disciplines (2024‐PWXZ‐06).

## Ethics Statement

This research was approved by the Ethics Committee of Shanghai Pudong New Area People's Hospital & Shanghai Pudong New Area Gongli Hospital (2025‐D‐96).

## Consent

All of the written informed consents had been acquired, and on behalf of all participants, we approved the publication of this research.

## Conflicts of Interest

The authors declare no conflicts of interest.

## Supporting information


**Figure S1:** A, Cell proliferation of A549 and PC9 cells treated with exosomes derived from vector or HMGB1 OE cells (cell‐to‐exosome ratio = 1:10), combining HMGB1‐IN1 (100 nM) or not. B, Cell migration of A549 and PC9 cells treated with exosomes derived from vector or HMGB1 OE cells (cell‐to‐exosome ratio = 1:10), combining HMGB1‐IN1 (100 nM) or not. C, Colon formation capability of A549 and PC9 cells treated with exosomes derived from vector or HMGB1 OE cells (cell‐to‐exosome ratio = 1:10), combining HMGB1‐IN1 (100 nM) or not.
**Figure S2:** A, A549 and PC9 cells were seeded at densities of 1 × 105, 1 × 106 and 5 × 106 cells per six‐well plate and cultured for 3 days. Exosome production was then analysed. B, mRNA level of TLR4 expression in A549 and PC9 treated with scramble or TLR4 siRNAs. C, Western blotting of NF‐κB in A549/PC9 cells treated with exosomal HMGB1, combined with scramble and TLR4 siRNAs. D, Immunofluorescence staining of p‐STAT3 of A549 and PC9 cells treated with exosomal HMGB1, combined with PBS or IL‐6 neutralising antibodies (0.5 μg/mL). E, Western blotting of p‐JAK2, t‐JAK2, p‐STAT3 and t‐STAT3 in A549 cells treated with exosomal HMGB1 for 0, 24, 48 and 72 h. F and G, THP‐1–derived M0 macrophages were treated with PBS, HMGB1 (10 ng or 100 ng) or exosomes derived from vector or HMGB1 OE cells (cell‐to‐exosome ratio = 1:10). M1 macrophage markers (CD86) and M2 markers (CD206) were quantified by western blotting. The TNF‐α and IL‐10 in the supernatant were quantified by ELISA. H, Human peripheral blood‐derived M0 macrophages were treated with PBS, HMGB1 (10 ng or 100 ng) or exosomes derived from vector or HMGB1 OE cells (cell‐to‐exosome ratio = 1:10). M1 macrophage markers (CD86, CD80, iNOS) and M2 markers (CD206, IL‐10, Arg1) were quantified by PCR.

## Data Availability

The data that support the findings of this study are available in the Supporting Information of this article.
