# Supplementary material for: Exosomal HMGB1 Orchestrates NSCLC Progression and Immunosuppressive Macrophage Polarisation Through the TLR4/NF‐κB/IL‐6/STAT3 Signalling Cascade
Source: J Cell Mol Med. 2026 Feb 6;30(3):e71050. doi: 10.1111/jcmm.71050 (PMC12877720; doi:10.1111/jcmm.71050)
Supplement: Supplementary file 1 — Figure S1: A, Cell proliferation of A549 and PC9 cells treated with exosomes derived from vector or HMGB1 OE cells (cell‐to‐exosome ratio = 1:10), combining HMGB1‐IN1 (100 nM) or not. B, Cell migration of A549 and PC9 cells treated with exosomes derived from vector or HMGB1 OE cells (cell‐to‐exosome ratio = 1:10), combining HMGB1‐IN1 (100 nM) or not. C, Colon formation capability of A549 and PC9 cells treated with exosomes derived from vector or HMGB1 OE cells (cell‐to‐exosome ratio = 1:10), combining HMGB1‐IN1 (100 nM) or not. Figure S2: A, A549 and PC9 cells were seeded at densities of 1 × 10^5^, 1 × 10^6^ and 5 × 10^6^ cells per six‐well plate and cultured for 3 days. Exosome production was then analysed. B, mRNA level of TLR4 expression in A549 and PC9 treated with scramble or TLR4 siRNAs. C, Western blotting of NF‐κB in A549/PC9 cells treated with exosomal HMGB1, combined with scramble and TLR4 siRNAs. D, Immunofluorescence staining of p‐STAT3 of A549 and PC9 cells treated with exosomal HMGB1, combined with PBS or IL‐6 neutralising antibodies (0.5 μg/mL). E, Western blotting of p‐JAK2, t‐JAK2, p‐STAT3 and t‐STAT3 in A549 cells treated with exosomal HMGB1 for 0, 24, 48 and 72 h. F and G, THP‐1–derived M0 macrophages were treated with PBS, HMGB1 (10 ng or 100 ng) or exosomes derived from vector or HMGB1 OE cells (cell‐to‐exosome ratio = 1:10). M1 macrophage markers (CD86) and M2 markers (CD206) were quantified by western blotting. The TNF‐α and IL‐10 in the supernatant were quantified by ELISA. H, Human peripheral blood‐derived M0 macrophages were treated with PBS, HMGB1 (10 ng or 100 ng) or exosomes derived from vector or HMGB1 OE cells (cell‐to‐exosome ratio = 1:10). M1 macrophage markers (CD86, CD80, iNOS) and M2 markers (CD206, IL‐10, Arg1) were quantified by PCR. [file JCMM-30-e71050-s001.docx]

**Supplementary information**


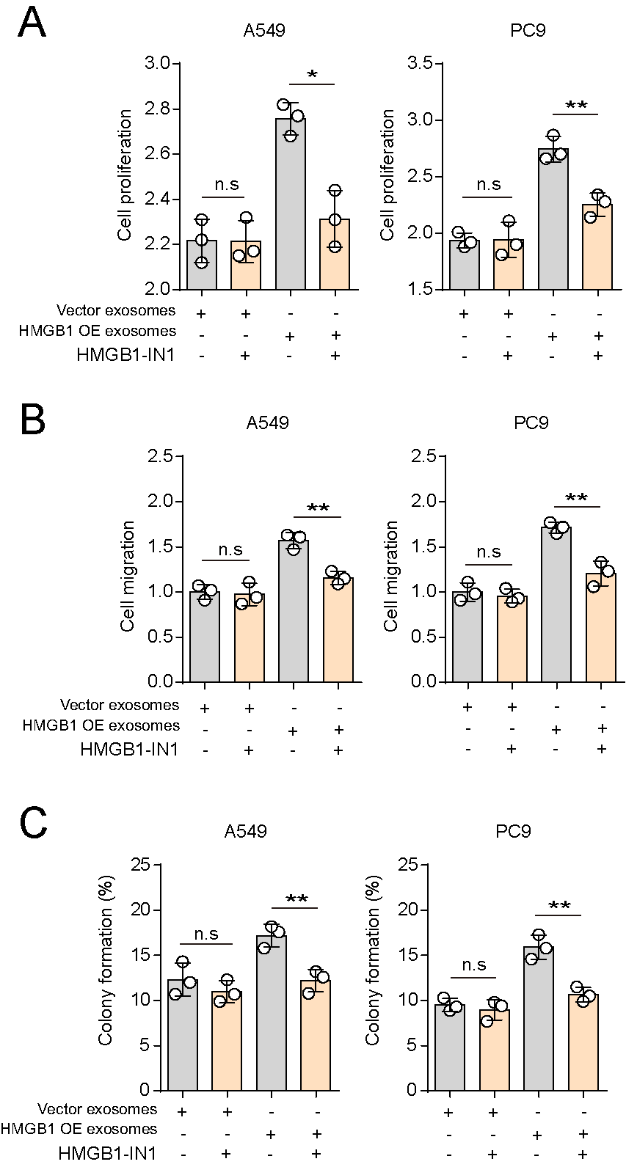


Supplementary figure 1

A, Cell proliferation of A549 and PC9 cells treated with exosomes derived from vector or HMGB1 OE cells (cell-to-exosome ratio = 1:10), combining HMGB1-IN1 (100 nM) or not. B, Cell migration of A549 and PC9 cells treated with exosomes derived from vector or HMGB1 OE cells (cell-to-exosome ratio = 1:10), combining HMGB1-IN1 (100 nM) or not. C, Colon formation capability of A549 and PC9 cells treated with exosomes derived from vector or HMGB1 OE cells (cell-to-exosome ratio = 1:10), combining HMGB1-IN1 (100 nM) or not.


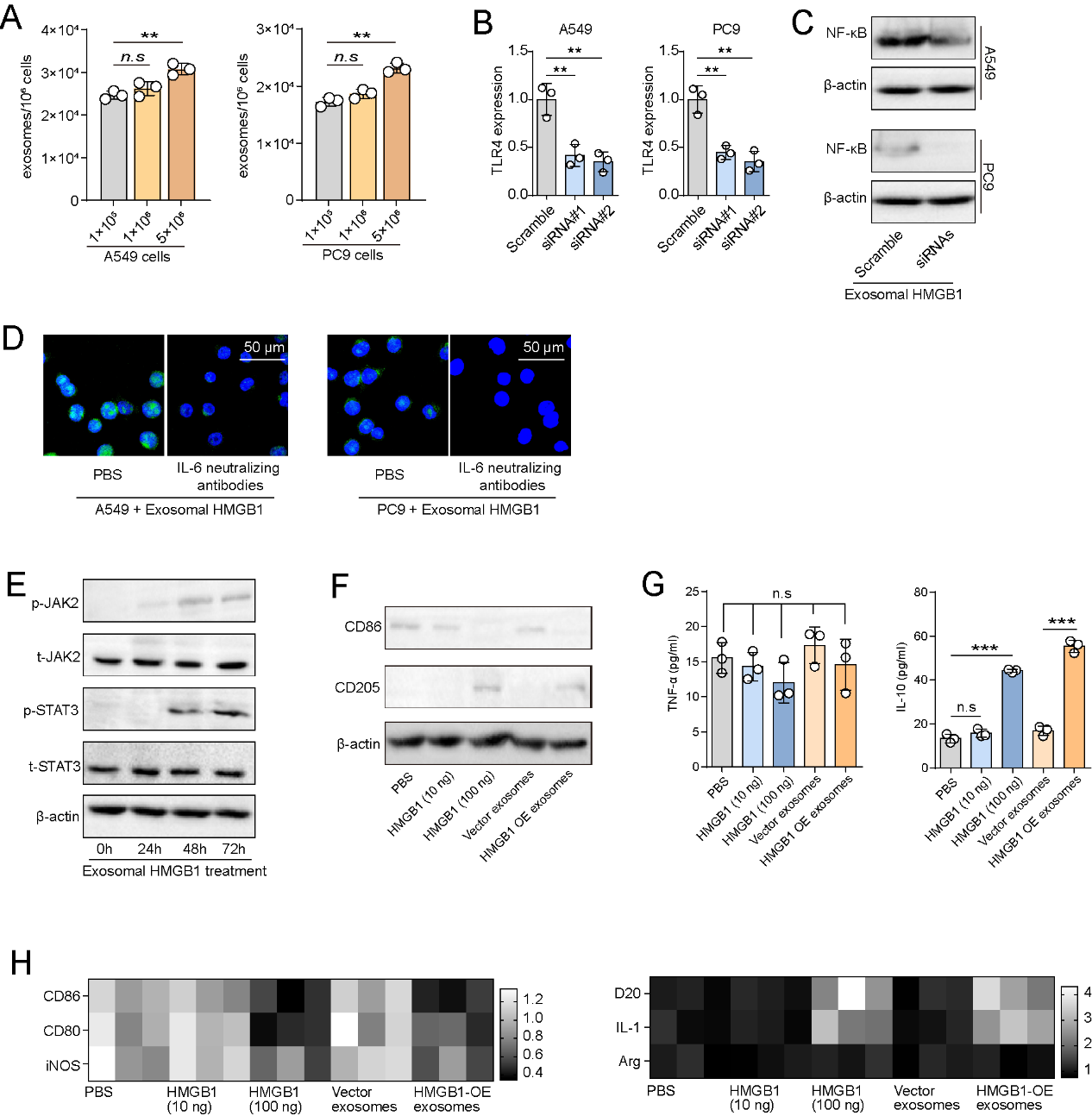


Supplementary figure 2

A, A549 and PC9 cells were seeded at densities of 1×10⁵, 1×10⁶, and 5×10⁶ cells per 6-well plate and cultured for 3 days. Exosome production was then analyzed. B, mRNA level of TLR4 expression in A549 and PC9 treated with scramble or TLR4 siRNAs. C, Western blotting of NF-κB in A549/PC9 cells treated with exosomal HMGB1, combined with scramble and TLR4 siRNAs. D, Immunofluorescence staining of p-STAT3 of A549 and PC9 cells treated with exosomal HMGB1, combined with PBS or IL-6 neutralizing antibodies (0.5 μg/ml). E, Western blotting of p-JAK2, t-JAK2, p-STAT3 and t-STAT3 in A549 cells treated with exosomal HMGB1 for 0, 24, 48, and 72 hours. F and G, THP-1–derived M0 macrophages were treated with PBS, HMGB1 (10 ng or 100 ng), or exosomes derived from vector or HMGB1 OE cells (cell-to-exosome ratio = 1:10). M1 macrophage markers (CD86) and M2 markers (CD206) were quantified by western blotting. The TNF-α and IL-10 in supernatant were quantified by ELISA. H, Human peripheral blood-derived M0 macrophages were treated with PBS, HMGB1 (10 ng or 100 ng), or exosomes derived from vector or HMGB1 OE cells (cell-to-exosome ratio = 1:10). M1 macrophage markers (CD86, CD80, iNOS) and M2 markers (CD206, IL-10, Arg1) were quantified by PCR.
